# Congenital Hypogonadotropic Hypogonadism during Childhood: Presentation and Genetic Analyses in 46 Boys

**DOI:** 10.1371/journal.pone.0077827

**Published:** 2013-10-24

**Authors:** Audrey Vizeneux, Aude Hilfiger, Jérôme Bouligand, Monique Pouillot, Sylvie Brailly-Tabard, Anu Bashamboo, Ken McElreavey, Raja Brauner

**Affiliations:** 1 Université Paris Descartes and Fondation Ophtalmologique Adolphe de Rothschild, Pediatric Endocrinology Unit, Paris, France; 2 Université Paris Sud and Assistance Publique-Hôpitaux de Paris, Hôpital Bicêtre, Service de génétique moléculaire, pharmacogénétique, hormonologie, Le Kremlin Bicêtre, France; 3 Human Developmental Genetics, Institut Pasteur, Paris, France; Institut Jacques Monod, France

## Abstract

**Background:**

The majority of the patients reported with mutations in isolated hypogonadotropic hypogonadism (HH) are adults. We analysed the presentation and the plasma inhibin B and anti-müllerian hormone (AMH) concentrations during childhood and adolescence, and compared them to the genetic results.

**Methods:**

This was a retrospective, single-center study of 46 boys with HH.

**Results:**

Fourteen (30.4%) had Kallmann syndrome (KS), 4 (8.7%) had CHARGE syndrome and 28 (60.9%) had HH without olfaction deficit nor olfactive bulb hypoplasia. Eighteen (39%) had an associated malformation or syndromes. At diagnosis, 22 (47.8%) boys were aged <one year, 9 (19%) 1–11 and 15 (32.6%) 11–17.6 years. They presented with micropenis (n = 32, 69.6%, including all those <one year), cryptorchidism (n = 32, 69.6%, unilateral in 8, bilateral in 24), and/or pubertal delay (n = 11). The plasma inhibin B concentrations were normal in 8 (3 KS including one CHARGE and 5 other HH), at the lower limit of the normal in 6 and decreased in 13 (48%) boys. The AMH concentrations were normal in 15 (6 KS including one CHARGE and 9 other HH) and decreased in 12 (44%) boys. In addition to the *CHD7* gene mutations in 4 patients with CHARGE, mutations were found in 5/26 other boys analysed including one in KAL1 gene with STS, 2 in *FGFR1* gene, one in *PROKR2* gene and one in *GnRHR* gene.

**Conclusions:**

The presence of micropenis in neonate, particularly if associated with cryptorchidism, is an indication to look for gonadotropin deficiency isolated or associated with other hypothalamic-pituitary deficiencies. Inhibin B and AMH concentrations are suggestive if low, but they may be normal. Despite the high frequency of the associated malformations and excluding the patients with CHARGE or ichtyosis, the 4 patients with mutations had no family history or malformation. This suggests that many other genes are involved.

## Introduction

Congenital hypogonadotropic hypogonadism may be isolated or associated with other hypothalamic-pituitary deficiencies. It is diagnosed in young boys when they are neonates or infants, or at pubertal age. Neonates and infants may present with micropenis and/or cryptorchidism. These signs can be isolated (isolated hypogonadotropic hypogonadism (HH)) [1] or associated with symptoms like hypoglycemia, jaundice and decreased growth rate in cases where hypogonadotropic hypogonadism is associated with other hypothalamic-pituitary deficiencies [2].

Boys in the pubertal age may present with pubertal delay and distinguishing HH from constitutional delay of growth and puberty may be difficult in this situation. A recent study evaluates the literature regarding the ability of diagnostic tests to distinguish between these two situations [3] and concluded that basal inhibin B may offer a simple, discriminatory test if results from two recent studies [4], [5] are replicated.

A genetic cause has been reported in around 30% of HH cases, with most gene mutations identified in association with Kallmann syndrome (KS) that includes HH, hyposmia or anosmia and frequently other malformations. Currently mutations in eight genes explain approximatively 25–35% of KS cases [6], [7]. In HH without olfaction deficit, mutations in six genes have been reported. Some of KS genes (such as fibroblast growth factor receptor 1 (*FGFR1), FGF8, PROKR2* and *PROK2* can also be associated with a normosmic HH. These mutations are associated with a variable transmission (mainly recessive transmission) and can be associated with various malformations and degrees of HH including reversibility [8].

The majority of the patients reported with mutations in HH are adults. We therefore analysed 46 boys seen by the same physician during the childhood or adolescence for HH. We describe their clinical presentation, associated malformations and the plasma inhibin B and antimüllerian hormone (AMH) concentrations, and compared them to the genetic analyses. We expect these results to help with the diagnosis and the management of young patients with HH.

## Materials and Methods

### Ethics statement

Signed informed consent for the evaluations was obtained from the children's parents and included in the children's hospital medical record. Signed informed consent was also given for the genetic studies. All clinical investigations were conducted according to the principals expressed in the Declaration of Helsinki. The Ethical Review Committee (Comité de Protection des Personnes, Ile de France III) approved this retrospective study and stated that “This study appears to be in accordance with the scientific principles generally accepted and to the ethical standards of research. The study was lead in the respect of the French law and regulation”.

### Patients

This retrospective single-center study was carried out on 46 consecutive boys monitored for HH by a senior pediatric endocrinologist (R Brauner) in a university pediatric hospital from June 1981 to June 2012 (over 31 years). The 8 boys with HH due to *DAX* mutation [9] or those with Prader Willi syndrome were not included, as well as 6 other boys with HH but insufficient clinical-biological data and 6 girls with HH. They were first seen as neonates or up to 17.6 years for micropenis, unilateral or bilateral cryptorchidism and/or pubertal delay.

Isolated HH was diagnosed by low or absent gonadotropin response to a gonadotropin hormone releasing hormone (GnRH) test; it was performed twice in 13 cases. We reported the results of the first test. The second test in these cases was performed at pubertal age to confirm HH. Their plasma testosterone remained low when checked during the follow-up after 14 years of age, including those whose samples were collected after testosterone treatment was stopped for one month every year, and when they stopped growing. We measured the fasting blood glucose and cortisol (08.00 h), free thyroxin and insulin-like growth factor 1 of all patients seen during the first year of life to identify and exclude hypothalamic-pituitary deficiencies other than HH. KS was diagnosed by hyposmia or anosmia, documented by olfactometry, and/or hypoplasia or aplasia of the olfactive bulbs on magnetic resonance imaging (MRI).

### Methods

The history of each patient, including consanguinity and familial forms of diseases, was recorded. Pre- and perinatal histories were reviewed. Pubertal development was rated according to Marshall and Tanner [10]. Micropenis was defined by a penis length of less than 30 mm [11], [12]. The evaluation included MRI of the hypothalamic-pituitary region and olfactive bulbs (n = 27), olfactometry (n = 16), and ultrasound evaluation of the kidneys in KS (n = 3) and in CHARGE (coloboma, heart defects, atresia choanae, retardation of growth and/or development, genitourinary problems, ear abnormalities combined with deafness, n = 1). Those patients seen before pubertal age for micropenis were given testosterone heptylate (100 mg/meter^2^ i.m. every 15 days, 3 times), which restored penis length to normal [1], [13].

Plasma hormone concentrations were measured using different immunoassays during the study period. A new assay method for a given hormone was always cross-correlated with the previous method. Thus, the results are comparable throughout the whole period. An aliquot of plasma was frozen at −20°C to measure inhibin B [14], [15] and AMH [16] in 27. Six boys, first seen at the onset of the study, were given i.m. injections of human chorionic gonadotropin (hCG, 3×1500 U every other day); plasma testosterone was measured before treatment and on the day after the last hCG injection.

Inhibin B was measured in serum by an enzyme immunometric assay (Oxford Bio-Innovation reagents, Serotec, Oxford, UK), as well as AMH (Immunotech reagents; Beckman Coulter Company, Marseille, France). The inter and intraassay coefficients of variation were <7% for inhibin B and 8.7% and 5.3% respectively for a serum AMH concentration of 35 pmol/L and 7.8% and 4.9% for a serum AMH concentration of 1100 pmol/L. Follicle-stimulating hormone (FSH) and luteinizing hormone (LH) were measured with a sensitive immunoradiometric assay (CIS bio international, GIF sur Yvette, France), with a detection limit of 0.05 IU/L in both assays (IU/L 2^nd^ IRP WHO 78/549 for FSH, IU/L 1 ^st^ IRP 68/40 for LH).

Results are expressed as mean±SD. Groups were compared with a Mann-Whitney U test.

### DNA analysis

The blood karyotype was performed in 14 and the Array-Comparative Genomic Hybridation analysis in 8 cases [17]. The mutational analysis was performed in 26 cases the DNA being unavailable in 16 patients (including 3 with KS) seen at the onset of the study. Mutations in the genes *GnRH1*, *GnRHR, KISS1, KISS1R, TAC3, TACR3, KAL1, FGFR1, PROKR2, PROK2* and *SEMA3A* were detected by direct Sanger sequencing of PCR products as described elsewhere [18]–[22]. Sequence variations were found on both strands and confirmed in a separate PCR analysis.

Four other cases with chromodomain helicase DNA binding protein 7 (*CHD7*) mutation in CHARGE were diagnosed elsewhere before the study.

## Results

Fourteen (30.4%) patients had KS, 4 (8.7%) had CHARGE syndrome and 28 (60.9%) had HH, without olfaction deficit nor olfactive bulb hypoplasia. Among the 14 patients with KS, 10 had aplasia or hypoplasia of olfactive bulbs, one had normal bulbs despite anosmia and 3 (cases 2,3 and 6) were not evaluated by MRI because they had a diagnosed familial form of KS.

### Presentation (Table 1)

**Table 1:** Characteristics of the boys with HH.

														
Case	Family history	Malformation	Age at	Micropenis	Cryptorchidism	Testis volume	LH basal	LH peak	FSH basal	FSH peak	Inhibin B	AMH	MRI	Diagnosis
		Syndrome	diagnosis, yr		L/R	mL	U/L	U/L	U/L	U/L	pg/mL	pmol/L	Olfactive bulbs	
1	3 maternal cousins: behavioral disorder	bilateral nystagmus,cerebellar syndrome, psychomotor delay,deafness,cleft palate,IUGR	birth	1	R	3.75	0.4	1.1	0.9	3.6	96	385	absent	KS
2	brother and mother: KS	club foot	birth	1	0	0.4	0.2		0.38		95	128	NA	KS
3	brother and mother: KS		birth	1	0	1.5	0.2	1.2	1.2	5.3	80	134	NA	KS
4			birth	1	L+R	NA	0.1	1.6	0.3	1.5	33	309	normal	HH
5		CHARGE	birth	1	L+R	NA	<0.2	0.89	<0.2	3.9	NA	NA	NA	CHARGE
6	3 paternal uncles: KS + ichtyosis	ichtyosis	0.1	1	0	NA	0.3		0.5		NA	NA	NA	KS: KAL1 mutation + STS
7	mother: medical interruption of pregnancy at 20 weeks gestation for IUGR, hydrops and not ovaries 46,XX		0.1	1	0	4	0.4	2.5	0.4	8.7	13	4	normal	HH
8		bilateral cataract	0.1	1	L+R	1.5	0.2	0.9	0.2	1.1	184	765	normal	HH
9			0.1	1	L+R	NA	0.57	3.5	1.6	16.5	NA	NA	NA	HH
10	brother: micropenis,	coloboma, cataract,microcephaly, axial hypotonia,peripheral hypertonia,deafness, malformation of the inner and external ear,aortic insufficiency,long finger	0.1	1	L+R	NA	<0.4	2.5	<0.4	3.1	NA	NA	normal	HH
11		oculomotor congenital apraxia	0.2	1	L+R	NA	0.4	0.5	0.4	0.6		580	normal	HH
12			0.2	1	L+R	NA	0.7	1.1	0.4	0.4	209	970	normal	HH:PROKR2 mutation
13	brother: cryptorchidism		0.2	1	L+R	NA	0.1	3.8	0.7	29	50	315	absent	KS
14			0.3	1	R	2	0.21	3	1.3	12	24	174	absent	KS; FGR1 mutation
15			0.4	1	0	NA	0.6		0.4		NA	NA	NA	HH
16			0.6	1	L+R	NA	0.38	1.2	<0.2	3.5	NA	NA	normal	HH
17			0.7	1	0	2	0.2		0.3		NA	NA	normal	HH
18			0.7	1	R	2	0.2	1.3	1	4.2	22	285	NA	HH
19			1	1	L+R	NA	0.5	1.1	0.4	2.1	48	660	normal	HH
20			1	1	L+R	0.9	0.2	3.7	0.56	13	NA	NA	absent	KS
21		strabismus	1	1	L+R	NA	0.5	3.7	0.4	0.4	NA	NA	NA	HH
22			1	1	L+R	5	0.4	1.7	0.4	4.2	15	113	normal	HH
23			2	1	L+R	NA	0.5	1.8	0.37	6.4	NA	NA	normal	HH
24		cleft lip and palate	2	1	0	1.5	NA	NA	NA	NA	85	431	NA	HH
25	brother: cryptorchidism and mother precocious ménopause		3.5	1	L+R	1.05	0.2	1.6	0.55	10.2	NA	NA	NA	HH
26		hypospadias	7	1	0	NA	0.5	0.5	0.7	0.7	NA	NA	NA	HH
27		microphtalmia, coloboma, semicircular canals anomalies,sleep apnea	7.5	1	0	<4	0.1	1.2	0.2	1.7	10	88	NA	CHARGE
28		behavioral disorder, deafness	8.2	0	L+R	2	0.4	nc	0.4	nc	22	68	NA	HH
29			9.3	NA	0	3.8	0.1	4.1	2.4	7.8	83	826	R absent	KS
30		spinal muscular atrophy	9.3	NA	L+R	NA	<0.2	0.43	<0.2	0.94	NA	NA	NA	HH
31	father: cryptorchidism		10.5	1	L	1.5	nc	1.5	nc	1.3	NA	NA	L hypoplastic	KS
32			11.1	0	R	2	<0.2	0.57	0.33	2.1	NA	NA	normal	KS
33			12.3	1	L	2	0.2	0.44	0.2	1	NA	NA	NA	HH
34			13.3	0	R	1.5	0.2	0.86	0.33	1.6	92	459	normal	HH
35		left congenital facial paralysis,left cophosis and right deafness, semicircular canals anomalies,cleft palate,bicuspid aortic valve	13,5	NA	L+R	NA	NA	NA	NA	NA	NA	NA	L absentR hypoplastic	CHARGE
36			14.6	1	L+R	2	0.2	0.67	0.33	2.9	32	82	absent	KS FGFR1 mutation
37			14.8	NA	0	8	1.4	8.5	2.8	4.4	194	515	absent	KS
38		CHARGE	14.9	0	L+R	3	NA	2.5	7	2.2	73	371	normal	CHARGE
39			15	0	L+R	2	0.2	0.4	0.5	2.1	110	737	NA	HH
40			15.2	0	L+R	3	0.2	0.9	0.2	1.3	56	525	normal	HH
41			15.2	1	0	3.25	0.2	0.74	0.27	3.9	32	366	hypoplasia	KS
42	father and paternal family: Steinert's desease, hexadactyly	agenesis of the median incisor,hexadactyly,hypospadias,absence of nasal bridge	16	0	0	7	1	13	<1	<1	257	191	NA	HH
43			16.2	0	L+R	3.75	0.2	5	0.3	3.5	NA	NA	NA	HH GnRHR mutation
44		congenital strabismus	16.8	1	L+R	1.7	0.2	1	0.2	2.2	14	39	NA	HH
45		transposition of great vessels	16.8	NA	0	NA	0.7	4.6	1.1	1.9	201	NA	normal	HH
46			17.6	0	R	3.75	<0.5	<0.5	<0.5	1.5	<10	75	absent	KS
														

Anosmia in cases 2,3,13,29,32.

L: left R: right; 1 yes, 0 no;IUGR intrauterin growth retardation.

Pertinent family history was found in 9 boys. Two are brothers (cases 2 and 3) and their mother also had KS.

Two cases were born prematurely, case 16 at 26 weeks and case 20 at 33 weeks of gestation. Case 1 had intrauterine growth retardation. Eighteen (39%) boys had associated malformations or syndromes. These are mainly ophthalmic (7 cases, 15,2%) including coloboma (n = 2, associated with cataract in case 10 and with microphtalmia in case 27 with CHARGE), cataract (n = 2), strabismus (n = 2), nystagmus (n = 1) and/or oculor congenital apraxia (n = 1). The other malformations are neurologic (n = 5), ears (n = 5), dental agenesia (median incisor) or cleft lip and palate (n = 4), heart (n = 3) and/or orthopedic (n = 4).

At diagnosis, 22 (47.8%) boys were aged less than one year, 9 (19%) between 1 and 11 years, and 15 (32.6%) 11 to 17.6 years. They presented with micropenis (n = 32, 69.6%, including all those aged less than one year), cryptorchidism (n = 32, 69.6%, unilateral in 8, 17.4%, bilateral in 24, 52.2%), and/or pubertal delay (n = 11, 24%). In 5 patients seen after the first year of age, information concerning the penis length was unavailable. Excluding these, 22 (54%) patients had micropenis and unilateral or bilateral cryptorchidism combined.

All boys had basal LH <1 U/L and peak LH after GnRH test <5 U/L, except case 42 (see below) and case 37 who had normal pubertal response to the GnRH test despite a lack of testosterone secretion and the absent of olfactive bulbs by MRI. The mean plasma basal FSH concentration in the patients with KS is significantly greater than in the other HH cases (P<0.05, no including CHARGE). Similarly, the mean FSH peak after the GnRH test (P = 0.014) was also evelated in this group of patients. Plasma testosterone concentrations are very low, below 0.5 ng/mL in all patients, but in case 42 (see below) and case 45 (testosterone 0.71 ng/mL). The hCG test showed no significant increase (cases 6, 18, 26 and 31) or only a partial increase of plasma testosterone concentrations (1.4 ng/mL in case 34 and 1.9 ng/mL in case 39).

Case 42 has an interesting phenotype. He has a family history of consanguinity, which is unusual in this series, Steinert's disease and hexadactyly in his father and paternal family. He presented with agenesis of the median incisor, hexadactyly and distal hypospadias. He was the only case that did not have either a micropenis or cryptorchidism. He had normal basal and peak LH levels and undetectable basal and GnRH stimulated FSH concentrations and plasma testosterone concentrations of 1.2 ng/mL.

### Inhibin B and AMH (Table 1 and Fig 1 and 2)

**Figure 1:**
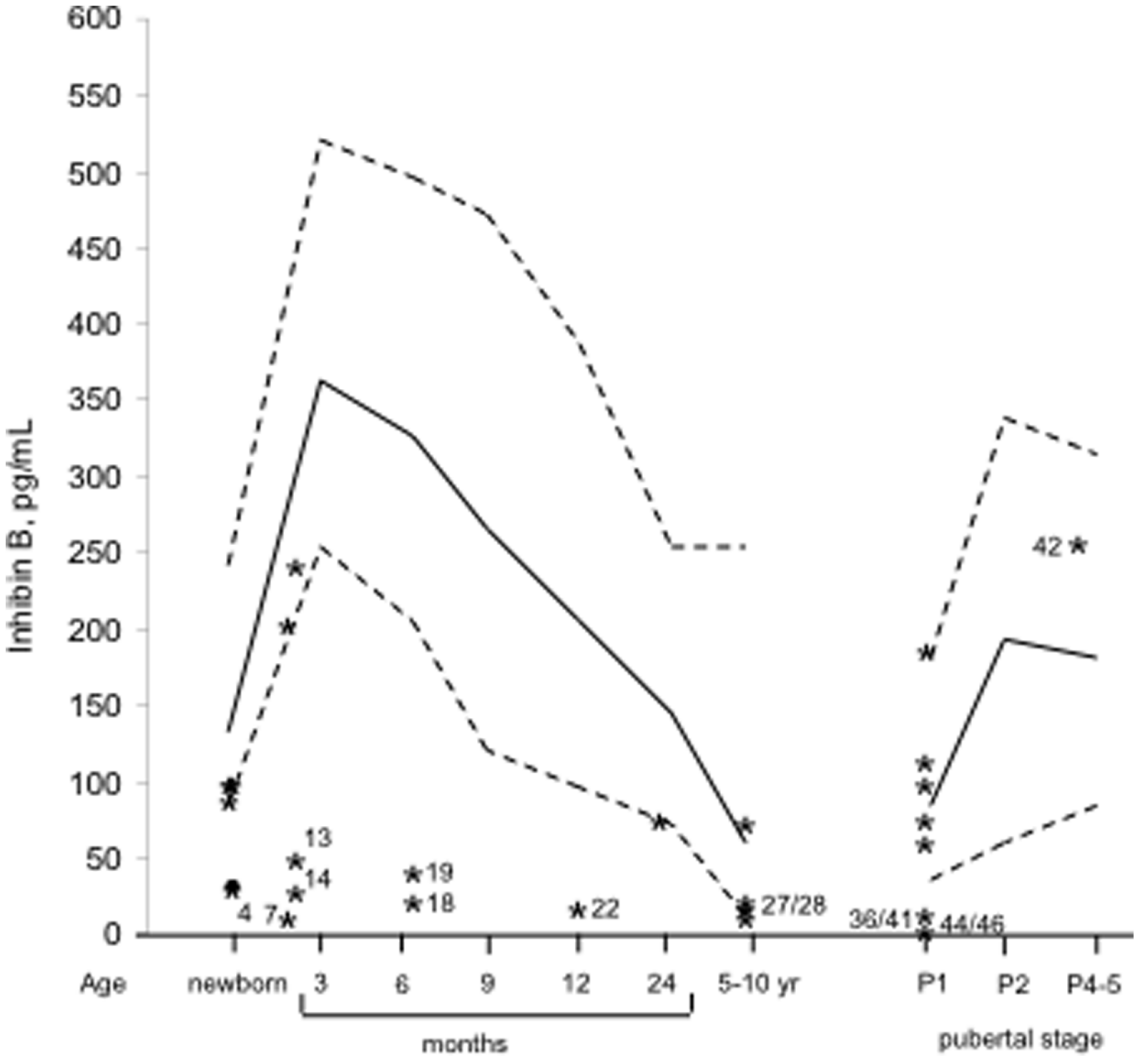
Distribution of the plasma inhibin B concentrations in 27 boys with isolated hypogonadotropic hypogonadism. Solid lines correspond to the median and broken lines to the 5^th^ and 95^th^ percentiles in infants [14] and at each stage of puberty [15]. Numbers (except 42) correspond to low concentrations (Tables 1 and 2): KS in cases 13,41 and 46, CHARGE syndrome in case 27, *FGFR1* mutation in cases 14 and 36 and other HH in the others.

**Figure 2:**
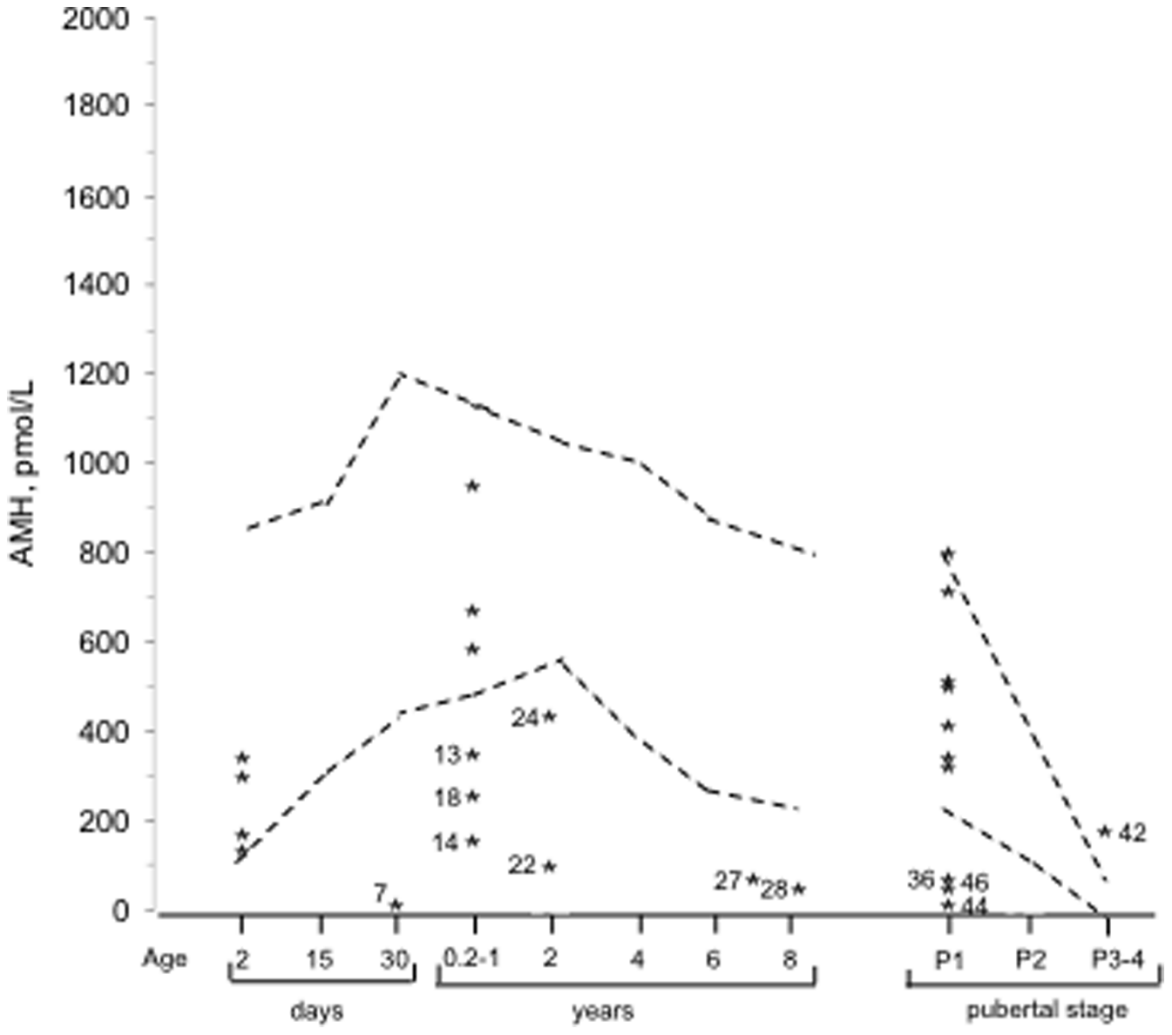
Distribution of the plasma AMH concentrations in 27 boys with isolated hypogonadotropic hypogonadism. Broken lines to the 5^th^ and 95^th^ percentiles [16]. Numbers correspond to low concentrations (Tables 1 and 2): KS in cases 13 and 46, CHARGE syndrome in case 27, *FGFR1* mutation in cases 14 and 36 and other HH in the others.

The plasma inhibin B concentrations were normal in 8 boys, including 6 in the pubertal age, at the lower limit of the normal in 6 boys and decreased in 13 boys. Among the 8 patients with normal inhibin B, 3 had KS including one with CHARGE syndrome, and 5 had other forms of HH. The plasma AMH concentrations were normal in 15 boys and decreased in 12 boys. Among the patients with normal AMH, 6 had KS including one with CHARGE syndrome, and 9 had other forms of HH. The concentrations of inhibin B and AMH are discordant in only 4 cases (low inhibin B but normal AMH in cases 4,19 and 41 and low AMH and inhibin B at the lower limit of the normal in case 24).

Both concentrations are low in the two patients with *FGFR1* mutation (cases 14 and 36), but normal in the patient with *PROKR2* mutation (case 12).

### Genetic analyses (Table 2)

**Table 2:** Genetic analyses.

														
Case	Karyotype	CGH Array	GnRH1	GnRHR	KISS1	KISS1R	TAC3	TACR3	KAL1	FGFR1	PROKR2	PROK2	SEMA3A	Diagnosis
1	46, XY		N				N	N		N				KS
2									N	N	N	N	N	KS
3	46, XY								N	N	N	N	N	KS
6			N	N			N	N	mutated					KS; KAL1 mutated,STS
7	46, XY		N	N	N	N	N	N		N	N	N		HH
8		N	N	N	N	N	N	N						HH
10	pericentric inversion of chromosome 9	N	N	N	N	N	N	N	N	N				HH
11		N	N	N	N	N	N	N						HH
12			N	N	N	N	N	N			mutated			HH: PROKR2 c.253C>T heterozygous missense mutation, p.Arg85Cys
13	46, XY		N	N	N	N	N	N	N	N	N	N	N	KS
14	46, XY								N	mutated				KS; FGFR1 c.286T>C heterozygous missense mutation, p.Ser96Pro. Mother N
17			N	N			N	N						HH
18			N	N										HH
19		N	N	N	N	N	N	N						HH
22			N	N	N	N	N	N						HH
23	46, XY	N	N	N		N								HH
25			N				N	N		N				HH
28	46, XY	N	N	N	N	N	N	N			N	N		HH
29									N	N	N	N	N	KS
31			N	N	N	N	N	N	N	N				KS
32									N	N	N	N		KS
36			N	N		N				mutated				KS; FGFR1 c.962_963delAA mutation; p.Lys321Argfs*13
40		N	N	N	N	N	N		N	N	N			HH
43			N	mutated	N	N	N	N						HH; R-GnRH Glu106Arg heterozygous mutation, c. 317 A>G. Mother N,
44			N				N	N		N				HH
45	46, XY	N												HH
46	46, XY										N	N		KS
														

Case 33: microduplication of chromosome 15, not found in the parents.

The karyotype, performed in 14 patients, was normal with the exception of a pericentric inversion of chromosome 9 in case 10 and a microduplication of chromosome 15 in case 33 (parents have a normal karyotype). Mutations were found in 5/26 (19.2%) boys analysed including one in the *KAL1* gene with steroid sulfatase isozyme S (*STS*), 2 in the *FGFR1* gene, one in the *PROKR2* gene and one in the GnRH receptor (*GnRHR*) gene. The patient with the *PROKR2* mutation has normal olfactive bulbs on MRI. His olfaction has not been evaluated (neonate).

## Discussion

This is the largest study reported on HH diagnosed during the childhood and adolescence. It shows that a micropenis and/or cryptorchidism were present symptom in all of the boys in whom the information was available except one. It also shows a high frequency of associated malformations (39%) with HH including the CHARGE syndrome in 4 boys. Excluding this syndrome, 4 out of 5 mutations were found in patients with an unexceptional family history.

### Presentation

In this study, micropenis and/or cryptorchidism were present in all of the boys in whom the information was available except one. The general prevalence of HH in the general population, based on civilian and military hospital series, is estimated 1/4000 to 1/10000 males [23]. The majority are discovered during adolescence or adulthood because of incomplete or absent pubertal development. Toublanc et al [24] reported that 84% of the males with late puberty had low plasma gonadotropins levels, among whom 31% had micropenis and 68% had cryptorchidism. Quinton et al [25] reported that 87% (7/8) patients of prepubertal age in a population of 170 males with HH, had micropenis and bilateral cryptorchidism. They reported that the overall prevalence of cryptorchidism was 50% and this was bilateral in 69% of the cases. Although 34% of the males in this series had undergone surgery for bilateral cryptorchidism during childhood, only 34% of these were referred for a pediatric endocrine investigation. Micropenis is the sign of a deficit in the secretion or in the action of the testosterone *in utero* between the 12^th^ week of amenorrhea and birth. The etiology of micropenis includes central hypogonadism (hypogonadism hypogonadotropic) or peripheral hypogonadism (hypogonadism hypergonadotropic), as well as resistance to androgens. Lee et al [26] attributed 31% of cases with a micropenis in the neonatal period to HH, another third of the cases remains “idiopathic”. Grumbach [27] insisted on the need to determine promptly whether the micropenis with or without cryptorchidism in an infant is due to isolated HH or HH associated with other hypothalamic-pituitary deficiencies as this is critical for reducing mortality and morbidity. The MRI can rule out pituitary stalk interruption syndrome and analyse olfactory bulbs, and the other anatomical structures. In patients with multiple hypothalamic-pituitary deficiencies due to pituitary stalk interruption syndrome, we found that all the boys with gonadotropin deficiency had micropenis and/or cryptorchidism [2]. Another emergency in a neonate with micropenis and with no papable testis is congenital adrenal hyperplasia, which, in rare cases induces the complete virilisation of female fetus. This can be excluded by the dosage of plasma concentration of 17-hydroxyprogestérone.

Malformations are associated with HH in 39% of the cases. Among the 20 boys with HH reported by Van Dop et al [28], 50% had cryptorchidism, 50% had ocular anomalies and 25% had skeletal anomalies.

### Inhibin B and AMH

Inhibin B and AMH are secreted by the Sertoli cells, stimulated by gonadotropins. Adult men with isolated HH have low plasma inhibin B concentrations [29]–[31]. These concentrations are lower in men with KAL [30], [32] and in those patients with the classic form of HH compared to men with the adult-onset form of HH [32], [33]. Pulsatile GnRH can restore these concentrations [29], [33]. Adult men with isolated HH have greater plasma AMH concentrations (292 pmol/L) than controls (20 pmol/L), which is similar to that of prepubertal boys [34].

In this study, the concentrations of inhibin B and AMH are frequently low, but they may be normal, including patients with KS. In 5/8 patients with normal inhibin B, these concentrations were compared to prepubertal values despite pubertal age. This suggests that the “normal” inhibin B concentrations are due to the absence of normal increase, which occurs at puberty and/or to a partial HH. Two studies evaluate the capacity of the plasma inhibin B and AMH concentrations to differentiate HH and constitutional delay of growth and puberty. Coutant et al [4] showed that 100% of boys with HH with genital stage 1, sensitivity and specificity had inhibin B concentrations of 35 pg/ml or less. They concluded that the discrimination of HH from constitutional delay of growth and puberty using inhibin B measurements was excellent in boys with genital stage 1 and fair in those with genital stage 2. We [5] found that these hormones may help differentiate HH and constitutional delay of growth and puberty, together with basal plasma concentrations of LH and FSH. The two studies showed that AMH concentrations performances are lower than that of inhibin B to distinguish between the two situations. As the increase of testosterone at puberty there is a corresponding decrease in the plasma AMH concentration [5]. The deficiency in gonadotropins decreases the plasma AMH levels, and a combination of no testosterone increase and gonadotropin deficiency in HH probably explains the difficulties in the interpretation of the plasma AMH concentrations.

### Genetic analyses

Currently, mutations in eight genes involved in the migration of GnRH neurons during embryonic development explains approximatively 25–35% of KS cases. These include the genes *KAL1*, *FGFR1, FGF8* which encodes the ligand of *FGFR1*, *PROKR2* and *PROK2*, *NELF* (nasal embryonic factor) and mutations in *CHD7* associated with the CHARGE syndrome. Semaphorin-3 A gene (*SEMA3A* on chromosome 7q21.11) another gene involved in neuronal migration has been recently reported in familial forms of KS [35]. Six out of 8 of these genes were studied in a subgroup of our patients. These analyses revealed mutation in *KAL1* associated with an *STS* mutation in one autosomic dominant form of KS with icthyosis, one mutation in *PROKR2* and 2 mutations in the *FGFR1* gene.

We found heterozygous *FGFR1* mutations in two boys, without a prior family history or malformation. These mutations are identified in approximatively 10% of individuals with KS, with variable phenotypic expression, usually in association with HH, cleft palate, mirror movements and dental agenesis. Trarbach et al [36] identified *FGFR1* heterozygous defects in 9/80 (11.2%) patients with sporadic or familial HH, including one with normal olfactory status. Among their patients with KS, the incidence of *FGFR1* mutation was 17% (28% familial and 8% sporadic). They found that prevalence of *KAL1* mutations in Brazilian male patients with KS is approximatively 22% (27% familial and 16% sporadic cases).

Mutations in *CHD7* are found in more than 60% of the patients with typical CHARGE syndrome. Conversely, mutations in *CHD7* are not a major cause of KS, because only 3–5% of patients with HH were found to have a *CHD7* mutation in two independent studies [37], [38]. Bergman et al [7] performed *CHD7* analysis in a cohort of 36 Dutch patients with KS that were previously excluded to carry mutations in *FGFR1, PROK2, PROKR2* and *FGF8*. They identified 3 heterozygous *CHD7* mutations. In these patients, additional features of CHARGE syndrome were found, the constant feature being bilateral hearing loss. They suggested that *CHD7* gene analysis should be performed in KS patients who have at least two of the following features of CHARGE syndrome: ocular coloboma, choanal atresia/stenosis, characteristic external ear anomaly, cranial nerve dysfunction, or balance disturbance.

In the HH without olfaction deficit, mutations in six genes have been reported. The most frequently reported mutations involve the genes encoding the *GnRH and Kisspeptin* receptors (*GnRHR* and *KISS1R*). Mutations in the *GnRHR* gene seem to be the most frequent cause of familial HH with normal olfaction, representing nearly 40% of cases in some series, but they are rarely found (<5%) in sporadic case [39]. We found a *GnRHR* gene mutation in one sporadic case. Mutations in *GPR54*/*KISS1R* are rare with only 10 other cases and one family reported by Nimri et al [40] with high frequency of consanguinity, no specific clinical faeture and a wide range of gonadotropin response to GnRH testing (from very weak or absent to relatively normal) even in subjects carrying the same mutation. We found no mutation in the *KISS1R* gene in the 9 patients without KS and 4 of these cases had an associated malformation, which has not been noted in the reported cases [40]. Mutations involving the genes *GnRH1* and *KISS1*, are a very rare cause of HH [41], [42]. Mutations in the *TAC3* and *TACR3* which code respectively for the neurokinine B (NKB) and its receptor (NK3R) were also identified in HH patients with a specific neuroendocrine profile: significantly higher mean FSH/LH ratio in 11 patients with *TAC3/TACR3* biallelic mutations than in other patients with HH [43]. Mutations in *FGFR1* and in *FGF8* genes have been reported in KS and in HH with normal olfaction, as well as those of *PROKR2* like in our patient with normal olfactive bulbs on MRI.

The case 42 presentation, detailed in Results, suggests isolated FSH deficiency, but the genetic analyses were not performed.

## Conclusions

The presence of a micropenis in a neonate, particularly if associated with cryptorchidism, is an indication to screen for a gonadotropin deficiency that may be isolated or associated with other hypothalamic-pituitary deficiencies.

The plasma concentrations of inhibin B and AMH are suggestive if low, but they may be normal including certain patients presenting with KS and who carry known gene mutations. The majority of the “normal” plasma inhibin B concentrations correspond probably to the absence of physiological pubertal increase in these concentrations and/or partial HH. The interpretation of normal plasma AMH levels is difficult, as the deficiency in gonadotropins decrease its concentration whereas the absence of testosterone increases its concentration. As inhibin B concentration is a marker of spermatogenesis, it may represent a pronostic marker of the fertility of these patients.

Despite the high frequency of the associated malformations and excluding the 4 patients with CHARGE or the one with ichtyosis, the 4 patients with mutations had no family history or malformation. This suggests that many other genes are probably involved.
